# Supplementary material for: Additive manufacturing–facilitated blow molding for functional thin-walled polymeric structures
Source: Sci Adv. 2026 Sep 18;12(38):eaeg3937. doi: 10.1126/sciadv.aeg3937 (PMC13588197; doi:10.1126/sciadv.aeg3937)
Supplement: Supplementary file 1 [file sciadv.aeg3937_sm.pdf]

Science Advances
AAAS

Supplementary Materials for

Additive manufacturing–facilitated blow molding for functional thin-walled polymeric structures

Junyu Chen *et al*.

Corresponding author: Dotan Ilssar, dilssar@ethz.ch; Dennis M. Kochmann, dmk@ethz.ch

Sci. Adv. **12**, eaeg3937 (2026)
DOI: 10.1126/sciadv.aeg3937

The PDF file includes:

Supplementary Text
Figs. S1 to S7
Legends for movies S1 to S6

Other Supplementary Material for this manuscript includes the following:

Movies S1 to S6

Supplementary Text

Fabrication and testing of hinges

The fabrication process of the hinge structure is illustrated in Figure S1A–C. The central axes of the parison and the mold are aligned during fabrication. After pressurization, the wall thickness varies as a result of the mold's radial geometry. Consequently, regions with smaller thickness exhibit reduced bending stiffness, enabling smooth rotational motion between the adjoining planes. All schematic diagrams shown here correspond to the convex hinge configuration in Figure 1c; the other two cases shown in Figure 1d,e follow a similar fabrication process.

To measure the rotational stiffness of different hinge designs, the experimental setup shown in Figure S1D was used. The blow-molded hinges with an axial length of 20 mm, marked in Figure S1D, were bonded to two rigid 3D-printed connectors. During testing, the specimen was mounted in an Instron 5943 universal testing machine, which applied controlled rotation at the top while measuring the resulting torque at the bottom.

Mechanical characterization of polypropylene (PP)

PP specimens for mechanical characterization were laser-cut according to the geometry specified in ASTM standard D638. Uniaxial tensile (Figure S2A) and relaxation (Figure S2B) tests were performed using an Instron 5943 universal testing machine at a displacement rate of 3 mm/min. During the relaxation test, the stress decreases over time at a fixed strain, indicating the viscoelastic behavior of the PP material.

Cross-section of the multistable conical shell

To preserve the cross-sectional geometry of the stable states, the multistable conical shell samples were first fixed onto 3D-printed holders in either the deployed or folded configuration and subsequently sectioned. This procedure ensures that the cross-sectional shape associated with each stable state is retained, which is not be possible if the samples are cut prior to fixation. Figure S3A shows the original cross-sectional micrographs of a multistable conical shell in its stable configurations. Figure S3B presents the corresponding images after background removal, retaining only the cross-sectional profile and highlighting the upper frustum in green.

Analytical prediction of the critical buckling force of a multistable conical shell

An analytical expression for the uniaxial force–displacement ($F - \zeta$) relation of a multistable conical shell was derived in (*37*) as

$$F \approx 8C_1\zeta\left(\zeta^2 - \zeta_0^2\right) + 2C_2(\zeta - \zeta_0), \tag{S1}$$

where C_1 and C_2 are coefficients determined by the material properties and geometric parameters:

$$C_1 = \frac{\pi E h}{8(R-r)^3}\left(\frac{R+r}{2} - \frac{R-r}{\ln(R/r)}\right), \tag{S2}$$

$$C_2 = \frac{\pi E h^3 \ln(R/r)}{12(R-r)^2}. \tag{S3}$$

A Young's modulus of $E = 1.7$ GPa was obtained from the secant modulus over the strain range of 0–0.5% in the uniaxial tensile test (Figure S2A). For the reference design, the frustum thickness is $h \approx 0.25$ mm, the outer and inner radii are $R = 11.25$ mm and $r = 8$ mm, respectively, the width is $w = R - r$, and the tilting angle is $\alpha = 36°$. The deflection of the initial folded stable state is approximated as $\zeta_0 \approx -(R - r)\,\alpha$. Figures S4a–c show the predicted dependence of the critical buckling force F_{cr} on the design parameters α, w, and R based on the theoretical model. These trends show qualitative agreement with the experimental results in Figure 2f–h of the main article.

Fabrication of laser-cut tubular kirigami

Tubular kirigami structures fabricated by laser cutting were produced by first cutting the prescribed kirigami pattern into a 0.2 mm thick polyethylene sheet (Figure S5A). The patterned sheet was then rolled into a tubular configuration and bonded to rigid connectors to form the final structure. This fabrication approach inherently introduces joint regions with double thickness at the bonded seam, as highlighted in Figure S5B, which can lead to local mechanical inhomogeneity.

Post-processing of blow-molded tubular kirigami structures.

After blow molding, the tubular kirigami structure was subjected to a simple post-processing procedure. The as-molded part was first trimmed, followed by polishing on sandpaper to remove the protrusions and form the slits that function as the kirigami cut patterns (Figure S6A,B). The processed kirigami tube was then attached to connectors (Figure S6C). Because the structure is

fabricated as a seamless tube, this procedure avoids the formation of joint seams or double-thickness regions, resulting in improved mechanical homogeneity.

Post-processing of blow-molded bellows for shape modification.

After blow molding, the bellow underwent a post-processing procedure to modify its geometry. The bellow was first placed in a custom fixture to constrain the desired shape (Figure S7A), followed by localized heating using a hot air gun to reform the structure. To ensure homogeneous heating, the assembly was rotated continuously during the heating process (Figure S7B). After cooling, the treated bellow retained the imposed compressed configuration without external constraint. Figure S7C compares bellows without and with heat treatment, demonstrating the effectiveness of this process in enabling shape modification and retention.

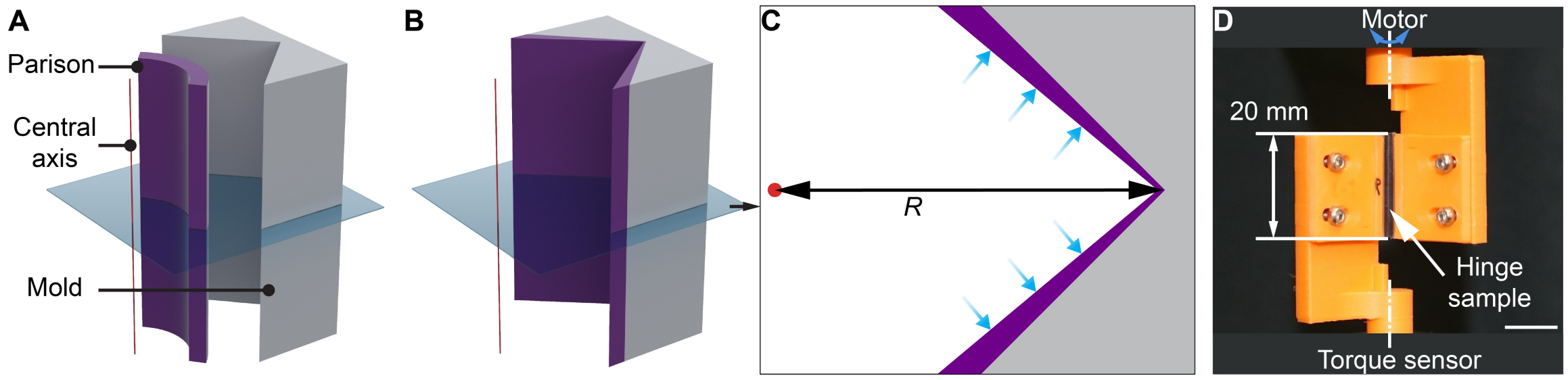

Figure S1: **Fabrication and testing of a hinge.** (A,B) 3D schematic illustrations of the hinge during fabrication: (A) before pressurization and (B) after pressurization. (C) Cross-section view of the hinge fabrication taken along the blue cutting plane. (D) Experimental setup for the torque measurement of the hinge samples (scale bar: 10 mm).

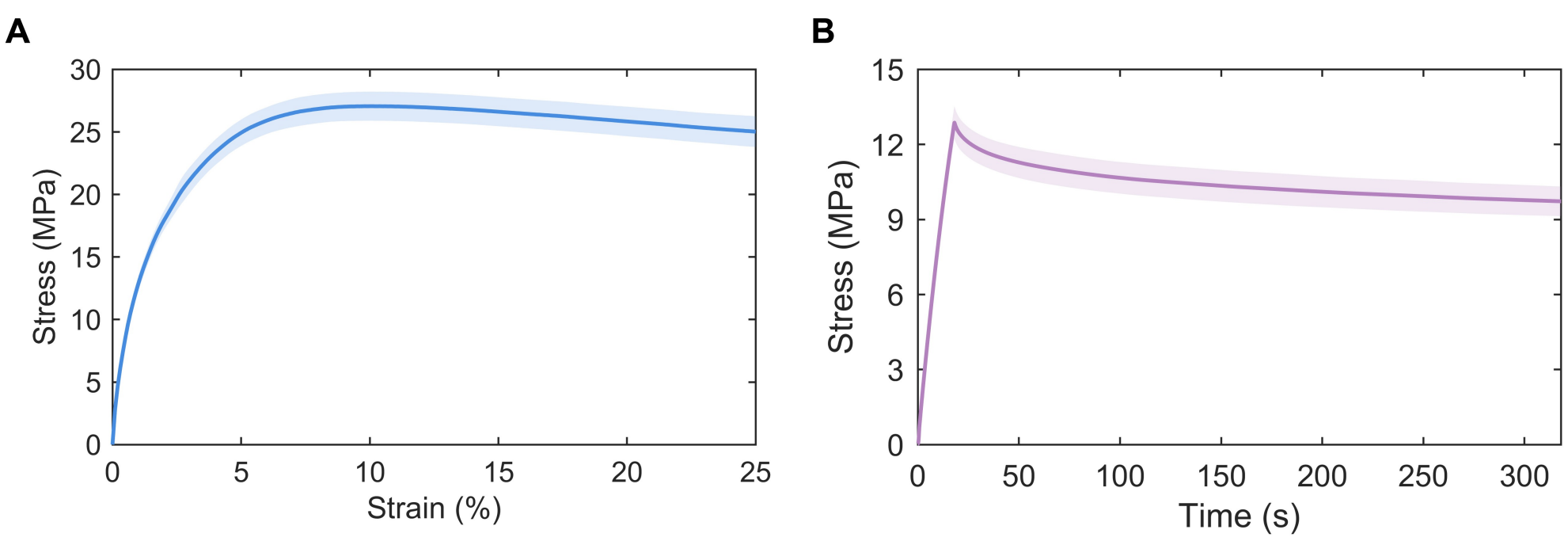

Figure S2: **Experimental results from mechanical tests on PP sample.** (A) Uniaxial tension test. (B) Stress relaxation test (shaded envelopes show standard deviations of three tests).

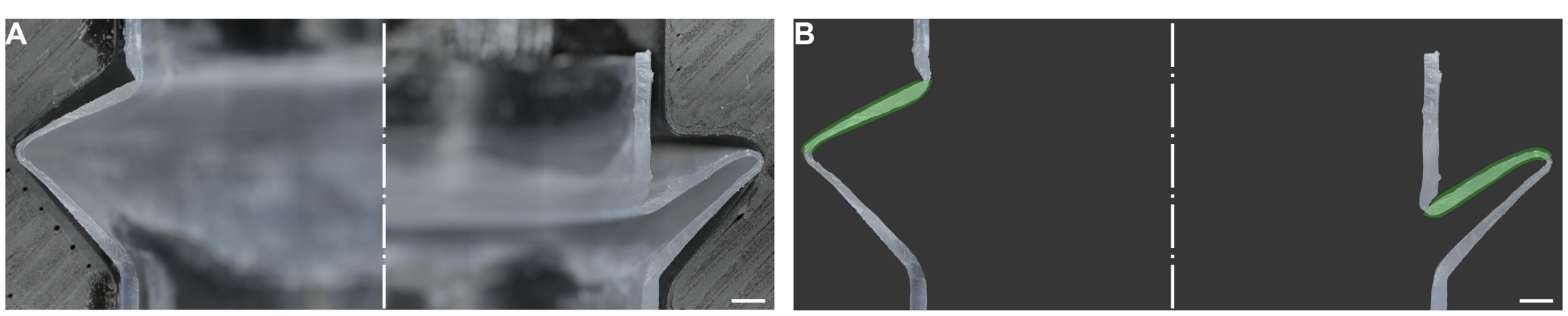

Figure S3: **Micrographs of the cross-section of a multistable conical shell in the deployed (left) and folded (right) configurations.** (scale bars: 1 mm) (A) Original micrograph without post-processing. (B) Post-processed image showing only the cross-section with the upper frustum highlighted.

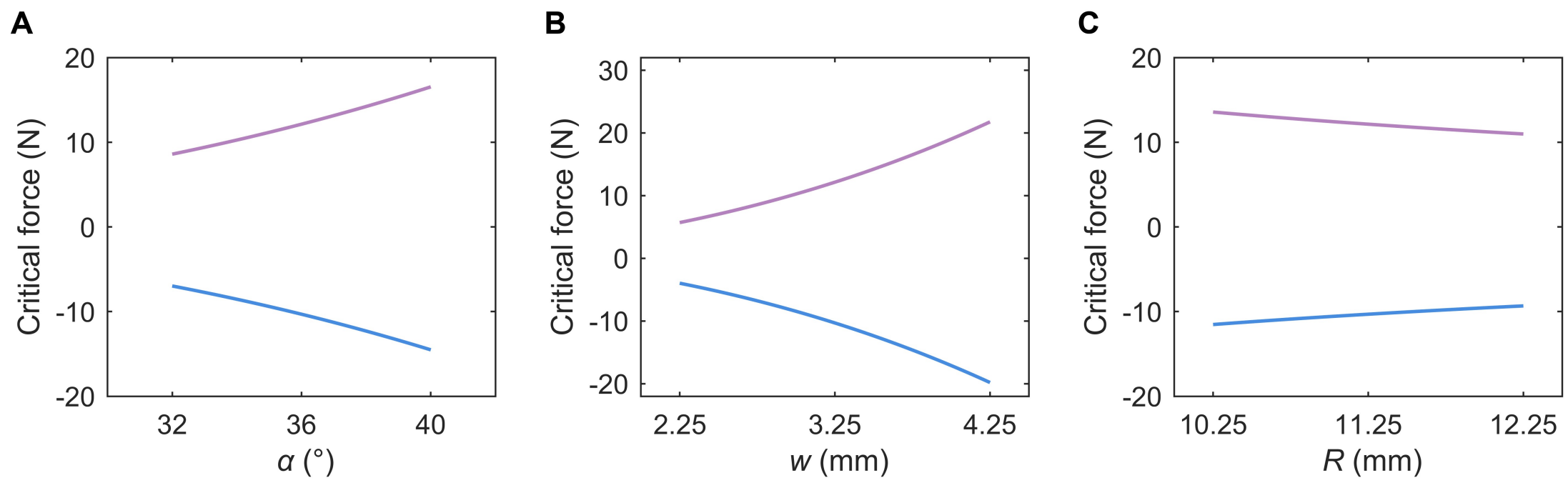

Figure S4: **Analytical prediction of the critical buckling force of a multistable conical shell.** Variation of the critical buckling force F_{cr} with design parameters of the conical shell: influence of (A) tilting angle α, (B) width w, and (C) outer radius R (purple and blue curves denote the critical buckling forces in extension and compression, respectively).

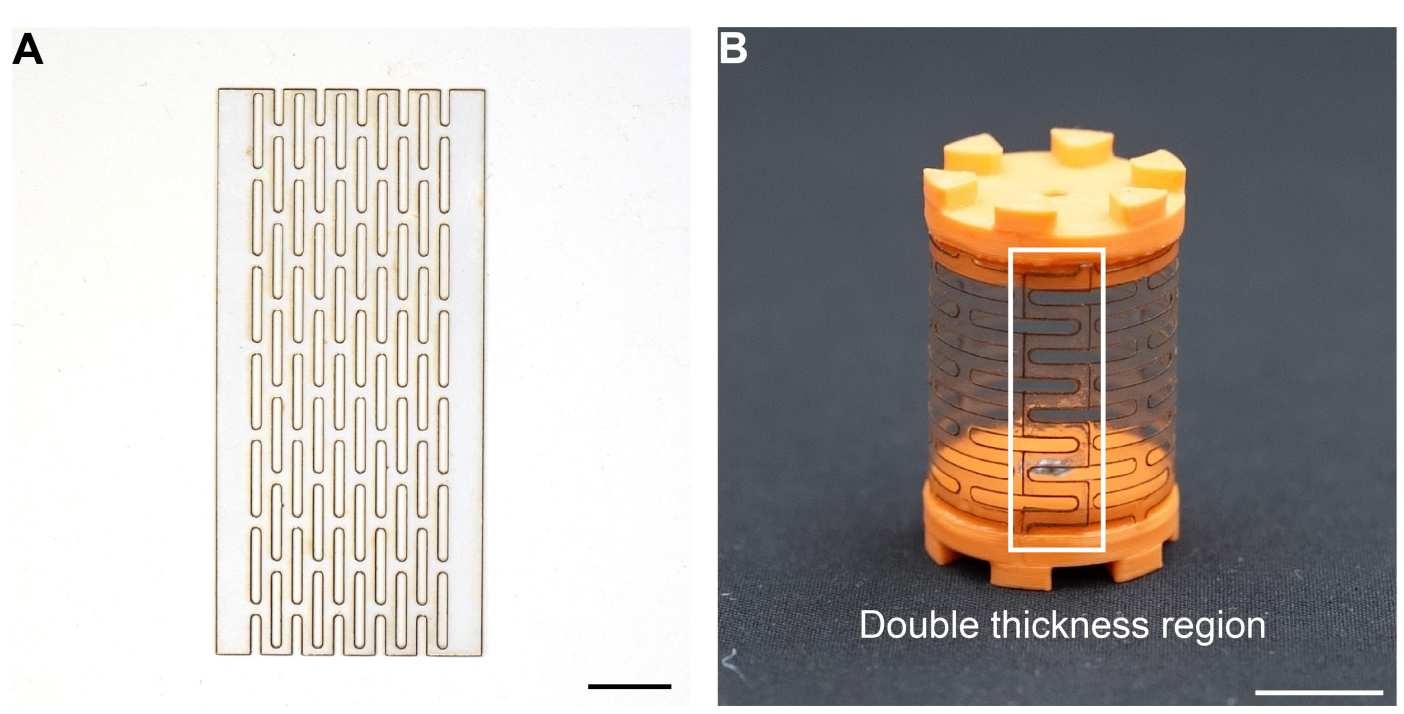

Figure S5: **Fabrication of laser-cut tubular kirigami.** (scale bars: 10 mm) (A) Laser cutting of the kirigami pattern on a polymer sheet. (B) Rolling the kirigami sheet into a tubular configuration and bonding it to connectors; the resulting double thickness region is indicated by the white rectangle.

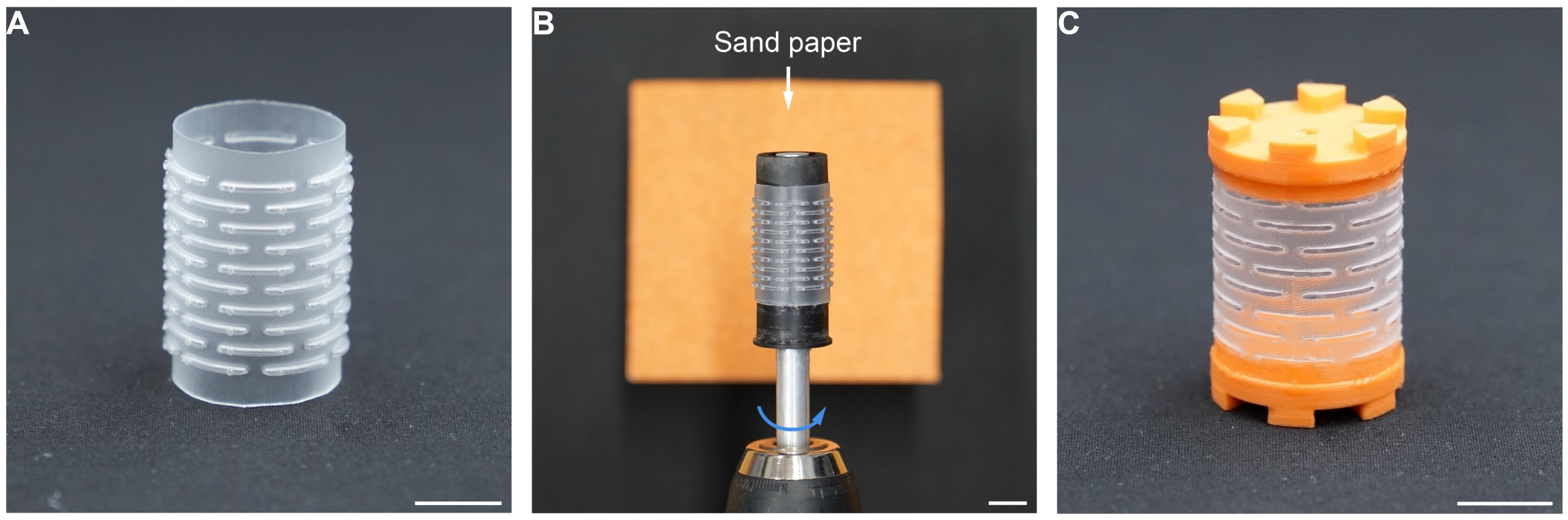

Figure S6: **Post-processing procedure for the blow-molded tubular kirigami structure.** (scale bars: 10 mm) (A) Trimmed part after blow molding. (B) Polishing on sandpaper to remove excess material. (C) Attaching the part to the connector.

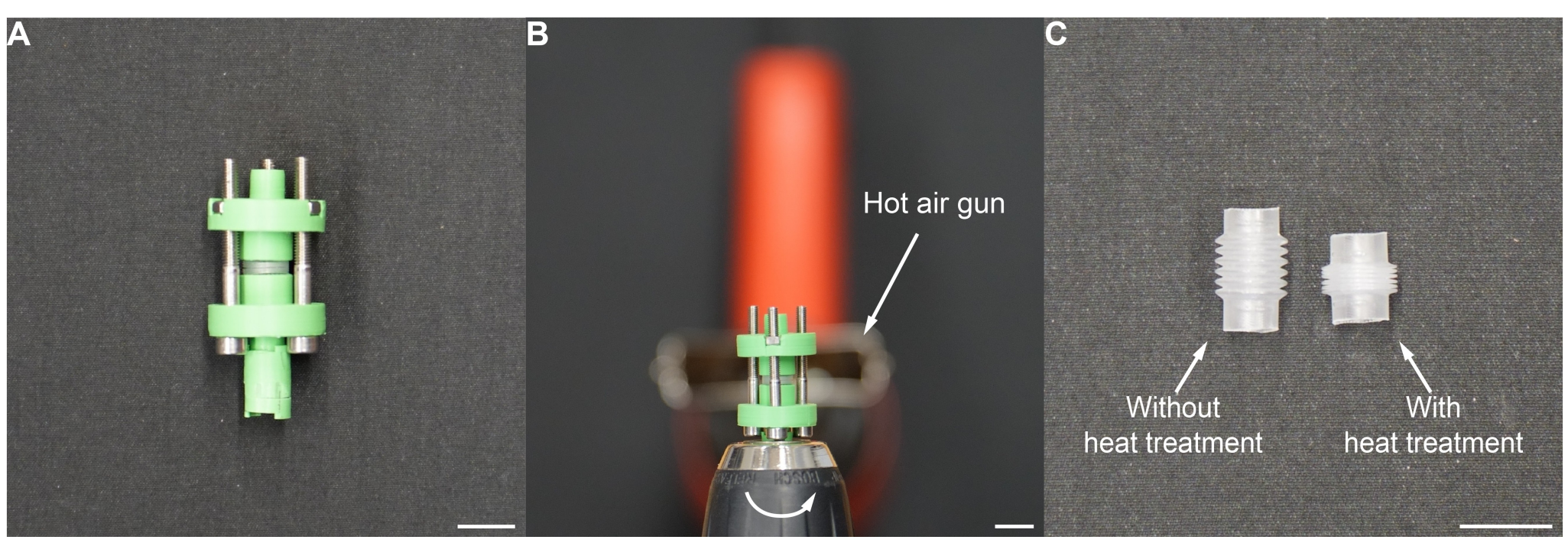

Figure S7: **Post-processing procedure for the blow-molded bellow.** (scale bars: 10 mm) (A) The bellow placed in a fixture. (B) Shape reformation using a hot-air gun. (C) Comparison between bellows without and with heat treatment after identical mechanical compression: both samples were compressed to the same displacement; the untreated sample (left) recovered its as-blow-molded shape upon unloading, whereas the heat-treated sample (right) retained the folded state.

Caption for Movie S1. Fabrication workflow of the AM-BM process. Preheated parison inflated inside a 3D-printed mold, followed by cooling and demolding to yield the final thin-walled structure.

Caption for Movie S2. Sequential reconfiguration of the multistable structure. A spatially graded straw transitions from the "00000" to the "10101" state using a single actuator, highlighting programmable multistability enabled by geometric design (animated version of Figure 2J).

Caption for Movie S3. Tubular kirigami patterns fabricated by AM-BM. Different origami tessellations—including Waterbomb, Kresling, Accordion, and Yoshimura patterns—exhibit diverse mechanical responses (animated version of Figure 3A-E).

Caption for Movie S4. Modified Yoshimura pattern operating as a pneumatic origami gripper. A modified Yoshimura origami pattern is able to achieve programmable directional bending and functions as a pneumatic gripper for objects of diverse shapes (animated version of Figure 3H-L).

Caption for Movie S5. Pneumatic artificial muscles with an exceptional load-to-weight ratio. The pneumatic actuator lifts a heavy load when pressurized, achieving a high load-to-weight ratio of 10,000 (animated version of Figure 4D-E).

Caption for Movie S6. AM-BM-fabricated modular soft robotic arm. The robotic arm demonstrates agile motion, open-loop trajectory following, and high load capacity (animated version of Figure 4F-I).
